# Item response theory analysis of Centers for Disease Control and Prevention Health-Related Quality of Life (CDC HRQOL) items in adults with arthritis

**DOI:** 10.1186/s12955-016-0444-4

**Published:** 2016-03-12

**Authors:** Thelma J. Mielenz, Leigh F. Callahan, Michael C. Edwards

**Affiliations:** Department of Epidemiology, Mailman School of Public Health, Columbia University, 722 West 168th St. Rm 512, New York, NY 10032 USA; Thurston Arthritis Research Center, School of Medicine, University of North Carolina, Chapel Hill, USA; Departments of Medicine and Social Medicine, School of Medicine, University of North Carolina, Chapel Hill, USA; Department of Psychology, The Ohio State University, Columbus, OH USA

## Abstract

**Background:**

Examine the feasibility of performing an item response theory (IRT) analysis on two of the Centers for Disease Control and Prevention health-related quality of life (CDC HRQOL) modules – the 4-item Healthy Days Core Module (HDCM) and the 5-item Healthy days Symptoms Module (HDSM). Previous principal components analyses confirm that the two scales both assess a mix of mental (CDC-MH) and physical health (CDC-PH). The purpose is to conduct item response theory (IRT) analysis on the CDC-MH and CDC-PH scales separately.

**Methods:**

2182 patients with self-reported or physician-diagnosed arthritis completed a cross-sectional survey including HDCM and HDSM items. Besides global health, the other 8 items ask the number of days that some statement was true; we chose to recode the data into 8 categories based on observed clustering. The IRT assumptions were assessed using confirmatory factor analysis and the data could be modeled using an unidimensional IRT model. The graded response model was used for IRT analyses and CDC-MH and CDC-PH scales were analyzed separately in flexMIRT.

**Results:**

The IRT parameter estimates for the five-item CDC-PH all appeared reasonable. The three-item CDC-MH did not have reasonable parameter estimates.

**Conclusions:**

The CDC-PH scale is amenable to IRT analysis but the existing The CDC-MH scale is not. We suggest either using the 4-item Healthy Days Core Module (HDCM) and the 5-item Healthy days Symptoms Module (HDSM) as they currently stand or the CDC-PH scale alone if the primary goal is to measure physical health related HRQOL.

## Background

Arthritis is the one of the leading causes of disability with an estimated 67 million US adults with doctor-diagnosed arthritis by 2030 [1]. People with arthritis are reported to have worse health-related quality of life (HRQOL) than people without arthritis [2, 3].

HRQOL has been measured among people with arthritis in several ways. The Medical Outcomes Survey 36-item and 12-item short-form surveys (SF-36 and SF-12) are the best known HRQOL measures [4, 5]. The Centers for Disease Control and Prevention (CDC) felt a less burdensome measure of HRQOL was needed for broad population use [6]. The 4-item Healthy Days Core Module (HDCM) was developed in a series of workshops held in 1991 and 1992 for the 1993 Behavioral Risk Factor Surveillance System [6].

In 1995, the CDC added 10 additional items creating two optional modules (including the 5-item Healthy Days Symptom Module (HDSM) and the 5-item Activity Limitation Module [7, 8]. The CDC HRQOL measures have been analyzed using classical test theory and are reported to have adequate to strong psychometric properties in a population with arthritis [2, 3, 8–11].

In 2006, Mielenz et al. reported a classical test theory analyses of the 4-item HDCM and the 5-item HDSM [8]. The goal of the Mielenz et al. (2006) paper was to combine the 4-item HDCM and the 5-item HDSM into one 9-item HRQOL measure using classical test theory analyses. A two-factor solution emerged producing two subscales made-up of the 4-item HDCM and 4 of the 5-item HDSM described in more detail in the methods section below: 1) a 5-item scale called CDC-HRQOL Physical Health Scale (the CDC-PH) and 2) a 3-item scale called CDC-HRQOL Mental Health Scale (the CDC-MH) [8].

The goal of the current research is to conduct item response theory (IRT) analyses of the items assessing the CDC-PH and CDC-MH scales separately. Based on these previous analyses, both scales were comprised of a mix of items assessing physical and mental health. We felt it would be most useful to isolate the items assessing each construct as previously reported and try to create separate IRT scores representing physical and mental health.

## Methods

### Sample

The methods for this study have been previously published and they are briefly summarized here [8]. A self-report cross-sectional survey including the CDC 4-item HDCM and the 5-item HDSM was mailed to 4183 patients with self-reported arthritis in the fall of 2002. The participants are drawn from two ongoing cohorts: the North Carolina Family Medicine Resource Network (NC-FM-RN) and the musculoskeletal database (MSK). The original study was approved by the University of North Carolina Institutional Review Board. This secondary analysis was approved by the Columbia University Medical Center Institutional Review Board.

The NC-FM-RN is a practice-based network devoted to research on chronic diseases in primary care [12]. At the time of enrollment, participants confirmed their approval to be contacted for future studies. Participants were selected for this study if they self-reported osteoarthritis, rheumatoid arthritis, fibromyalgia, or reported any symptoms of pain, aching, or stiffness in or around a joint in the past thirty days. Out of the 4760 participants enrolled in the NC-FM-RN, 2182 were selected for this study based on these criteria. The MSK database enrolls consecutive adult patients from rheumatology (both academic and community clinics) and orthopedics. Participants in the MSK database can consent to be contacted for future studies. Participants (*n* = 2001) with osteoarthritis, rheumatoid arthritis and fibromyalgia were selected.

A total of 1820 participants completed surveys (1139 from the NC-FM-RN and 681 from the MSK). Details about the non-respondents and this response rate are published; a total of 631 participants were removed from the denominators of each sample due to incorrect addresses (*n* = 584) and deceased participants (*n* = 47) [8]. The response rate was 51 % when combining the completed surveys from both cohorts and this corrected denominator.

### Behavioral risk factor surveillance system HRQOL

The HDCM includes: 1) Would you say that in general your health is :[five responses ranging from excellent to poor], 2) Now thinking about your physical health, which includes physical illness and injury, for how many days during the past 30 days was your physical health not good? [the number of days in the past 30], 3) Now thinking about your mental health, which includes stress, depression, and problems with emotions, for how many days during the past 30 days was your mental health not good? [the number of days in the past 30], 4) During the past 30 days for about how many days did poor physical or mental health keep you from doing your usual activities, such as self-care, work or recreation? [the number of days in the past 30], [From http://www.cdc.gov/hrqol/hrqol14_measure.htm; Access date 02/15/16] The HDSM asks: During the past 30 days, for about how many days 1) did PAIN make it hard for you to do your usual activities, such as self-care, work, or recreation?, 2) have your felt SAD, BLUE, or DEPRESSED?, 3) have you felt WORRIED, TENSE, or ANXIOUS?, 4) have your felt you did NOT get ENOUGH REST or SLEEP?, 5) have you felt VERY HEALTH AND FULL OF ENERGY? With the exception of the full of energy item, higher scores indicate poorer health. The full of energy item was reversed scored for all analyses.

### CDC-PH and CDC-MH

The CDC-PH includes these five items described above: HDCM 1), 2) and 4) and HDSM 1) and 5) [8]. The CDC-MH includes the these three items described in the above section: HDCM 3) and HDSM 2) and 3). One item from the HDSM was dropped: During the past 30 days, for about how many days 4) have your felt you did NOT get ENOUGH REST or SLEEP [8]?

### IRT analyses

IRT is a set of models that describe the process by which individuals respond to items. Put another way, IRT is analogous to a factor analysis where the relationships between the measured variables and the latent construct are nonlinear [13]. One of the most widely used IRT models is the graded response model (GRM), which is appropriate when the responses are ordered categories [14]. The GRM has two types of parameters: discrimination and thresholds. The discrimination parameter provides information regarding how related each item is to the construct being measured. The threshold (or severity) parameters convey information related to the level of the underlying trait an individual would have to possess to choose any particular category with some probability. These parameters allow for the differential weighting of item responses when computing scale scores. The GRM is typically represented as:

$$ P\left({x}_j=c\left|\theta \right.\right)=\frac{1}{1+ \exp \left[-{a}_j\left(\theta -{b}_{cj}\right)\right]}-\frac{1}{1+ \exp \left[-{a}_j\left(\theta -{b}_{\left(c+1\right)j}\right)\right]} $$

where *x*_*j*_ is the observed response to item *j*, *c* is the particular response among the *C* response alternatives that was chosen, *θ* is the latent construct (singular in this case) being measures, *a*_*j*_ is the discrimination parameter for item *j*, and *b*_*cj*_ is one of the threshold parameters. For completeness we note that the first threshold is assumed to be minus infinity and the last infinity. This is a definitional part of the model and does not directly impact the estimated parameters. All IRT analyses in this paper were conducted using flexMIRT [15].

Some of the advantages of IRT include: 1) detailed item level information, 2) more accurate estimates of precision of individual scores, 3) item parameters that are not sample dependent, and 4) IRT is the segue into computerized adaptive testing [16]. Unfortunately, the CDC HRQOL scales pose a number of potential problems if the goal is to obtain item parameters via IRT modeling. First, the scales are relatively short, which has been shown to increase the difficultly in recovering parameters [17]. Next, as currently scored, there are a total of 31 response categories. While it is theoretically possible to use IRT with this many categories, it is rarely, if ever, done in practice. Lastly, when examining the questions, it seems possible that there could be local dependence among the items. Briefly, local dependence occurs when two (or more) items are more related to one another than the model would predict. Considered together, these impediments may preclude any IRT analysis of the CDC HRQOL scales. However, given the nature of our concerns weighed against the benefit of using an IRT approach, we decided to fit the models and then evaluate the stability/reasonableness of the resulting estimates.

### Natural response scale and IRT analysis

The natural response scale for the HDCM and HDSM is number of the past 30 days that a statement was true. This leaves 31 possible response categories. An analysis of the observed responses suggests that the bulk of respondents are using far fewer than 31 categories. For example, roughly 72 % of the responses for the second item on the HDCM fell into one of seven categories (0,5,10,15,20,25,30). Similar trends were found in the other items considered here as well. We chose to recode the data into eight categories which, along with the original observed frequencies, are described in Table 1. Our recoding scheme acknowledges the observed clustering, but also adds an additional category for those who are greater than zero, but less than five.

**Table 1:** Observed Frequencies and recoding scheme for the HDCM and HDSM

Freq	HDCM 2	HDCM 3	HDCM 4	HDSM 1	HDSM 2	HDSM 3	HDSM 4	HDSM 5
0	339	588	761	469	580	456	327	654
1	45	56	43	49	80	66	26	32
2	91	123	82	97	136	131	87	68
3	86	83	55	71	95	102	72	56
4	67	44	41	51	44	52	50	35
5	123	128	98	91	129	147	109	124
6	25	21	15	17	19	24	29	16
7	43	38	33	28	37	35	38	26
8	26	13	18	14	20	12	25	17
9	7	4	6	6	5	2	4	5
10	143	137	106	120	137	142	157	138
11	0	1	3	2	5	1	0	1
12	13	12	10	13	11	9	26	9
13	1	3	4	3	2	2	2	4
14	24	13	17	10	7	11	14	6
15	141	117	99	103	106	118	134	126
16	5	2	3	0	2	2	2	4
17	3	1	3	1	1	3	3	1
18	11	4	5	8	4	5	6	6
19	1	1	1	0	0	0	2	3
20	155	113	105	133	93	106	165	98
21	10	4	2	6	3	1	9	7
22	5	3	3	1	3	8	4	3
23	2	1	1	5	2	1	5	8
24	3	4	5	3	0	1	1	6
25	54	50	54	64	47	45	95	91
26	4	2	5	4	4	2	2	13
27	5	3	2	8	5	2	3	18
28	15	16	6	14	6	14	15	44
29	4	6	3	2	4	4	1	12
30	315	177	185	385	177	264	366	107
Missing	54	52	46	42	56	52	41	82

HDCM items 2-4 = 4-item Healthy Days Core Module (HDCM 1: self-rated global health with five responses is not shown here), HDCM 2: Physical health not good, HDCM 3: Mental health not good, HDCM 4: Poor health; HDSM = 5-item Healthy Days Symptoms Module, HDSM 1: Pain limited activities, HDSM 2: Depressed, HDSM 3: Stress HDSM 4: Not enough rest, HDSM 5: Full of energy (reversed scored)

### Assumptions of IRT analysis

For the kind of IRT analysis described in this paper, a critical assumption is that of unidimensionality. If a scale is unidimensional, then responses to that scale arise from only one underlying trait. A closely related assumption is that of local independence. Local independence implies that, conditional on the latent trait being measured, item responses are independent from one another. The unidimensionality and local independence assumptions can be assessed using confirmatory factor analysis (CFA).

The CFA models were estimated in LISREL using polychoric correlations and diagonally weighted least squares (DWLS) [18]. Using the DWLS estimator allows us to obtain correct fit indices such as Root Mean Square Error of Approximation (RMSEA) and the Comparative Fit Index (CFI) in the presence of categorical data [19]. CFI values greater than 0.95 are generally regarded as indicating good model fit. Browne and Cudeck (1993) characterize RMSEA values less than 0.05 as indicating close fit, values greater than 0.05 but less than 0.08 indicating reasonable fit, values greater than 0.08 but less than 0.1 indicating mediocre fit, and values greater than 0.1 indicating unacceptable fit [19, 20].

After listwise deletion, the sample size for the CFA analyses was *N* = 1642. We began by fitting a one factor model to all nine items to see if this simple model could account for the relationship among the items. As expected, this model did not fit the observed data well. The RMSEA for the 1-factor model was 0.24 and the CFI was 0.88, both of which suggest poor model fit. We subsequently fit a 2-factor model that was based on the results of the Mielenz et al. (2006) principal components analysis [8]. We started using all nine items, allowing five items (general health, physical health, physical/mental health, pain, energy) to load on a first factor, three items (mental health, depressed, anxious) to load on a second factor, and a single item (rest) to load on both. This model provided a reasonable fit to the observed data (RMSEA = 0.056, CFI = 0.99). The item about rest had significant, but weak (~0.35) factor loadings on both factors. This means that although the item is related to both factor, it does not provide a large amount of information regarding respondents levels on either factors. To retain this item for the IRT analyses would require the use of advanced multidimensional models which did not seem warranted given the limited information the item provides. We decided to remove that item and re-run the 2-factor model on the remaining eight items. This model had a similar level of fit as the previous model (RMSEA = 0.058, CFI = 0.994), which suggests that it provides an adequate representation of the data. All items had significant and sizeable (>0.7) factor loadings on their respective items. The two factors, which we labeled physical and mental health, were correlated 0.67. This is a large correlation, but not so large to suggest that there aren’t two unique constructs being measured by the eight items. These results match with a reading of the content of the items. Perhaps the only surprising result is the extent to which the item regarding physical and mental health limiting activities seems to have no relationship to the mental health factor. The data suggest that respondents are more heavily weighting their physical health when responding to this item, as it is predominantly related to other items assessing physical health. We also examined each dimension separately to assess model fit. The physical health scale fit reasonably (RMSEA = 0.08 and CFI = 0.99) and the mental health scale fit perfectly as that model is saturated with only three indicators.

## Results

### IRT analysis

After the factor analyses described above, we were confident that the data could be modeled using a unidimensional IRT model. The CDC-MH and CDC-PH scales were analyzed separately in flexMIRT. Multidimensional calibrations were also conducted in flexMIRT and the impact on the estimated parameters was negligible, which is not surprising given the particular structure of these data. The estimated correlation between the two dimensions was 0.65, consistent with the CFA findings. The estimation procedure used in flexMIRT is able to accommodate missing data without resorting to list-wise (or pair-wise) deletion, so we did not have to remove participants who had missing data. This resulted in a sample size of *N* = 1790 for subsequent IRT analyses.

The general health item, which is the first item on the CDC-PH, has five response categories. All other items, in both the CDC-PH and CDC-MH, have eight response categories as detailed above. The GRM produces one fewer threshold parameter than number of categories, which means there are four threshold parameters for the general health item and seven threshold parameters for all remaining items. Each item also has a slope parameter, which in the GRM is allowed to freely vary over items. The parameter estimates for the mental and physical health items can be found in Table 2. All item-level marginal χ^2^ values were non-significant and both models had an IRT-based RMSEA value of 0.05.

**Table 2:** Parameter estimates from the GRM for the CDC-Physical and Mental Health

Item description	a	b_1_	b_2_	b_3_	b_4_	b_5_	b_6_	b_7_
CDC-physical health								
HDCM 1:	2.24	−2.35	−1.05	0.12	1.51	-	-	-
General health								
HDCM 2:	3.52	−0.98	−0.44	−0.06	0.24	0.52	0.86	1.04
Physical health not good								
HDCM 4:	2.77	−0.27	0.08	0.38	0.66	0.92	1.24	1.49
Poor health								
HDSM 1:	2.97	−0.74	−0.27	0.01	0.26	0.47	0.75	0.94
Pain limited activities								
HDSM 5:	1.73	−2.2	−1.34	−0.97	−0.61	−0.27	0.10	0.47
Full of energy^a^								
CDC-Mental Health								
HDCM 3:	4.10	−0.47	0.01	0.34	0.64	0.89	1.18	1.4
Mental health not good								
HDSM 2:	7.71	−0.44	0.05	0.38	0.67	0.89	1.13	1.32
Sad, blue, depressed								
HDSM 3:	3.45	−0.71	−0.11	0.25	0.54	0.78	1.03	1.19
Worried, tense, anxious								

*GRM* graded response model, *HDCM* 4-item Healthy Days Core Module (# days except for HDCM 1), *HDSM4* 5-item Healthy Days Symptoms Module omitting the rest item (# days)

^a^HDSM 5 Full of energy was reversed scored for all analyses

A primary interest in initially examining the item parameters in Table 2 is assessing the extent to which the results appear reasonable. While there are five items proposed to tap into physical health (CDC-PH), there are only three relating to mental health (CDC-MH). In the factor analytic framework (of which IRT is a special extension) a latent variable with three indicators is just identified statistically. This can lead to issues with stability of estimation or lead to latent variables that are very narrowly construed. The parameter estimates for the five CDC-PH items all appear reasonable and within the boundaries commonly seen. The slope for HDSM2, which is a CDC-MH item, is unusually large. This could indicate that the solution is not stable, or it could indicate that responses to this one item capture most of the variability in responses to all three. While we are confident in the quality of the estimates for the CDC-PH items, we advise caution in using and/or interpreting the results for the CDC-MH items.

**Fig. 1:**
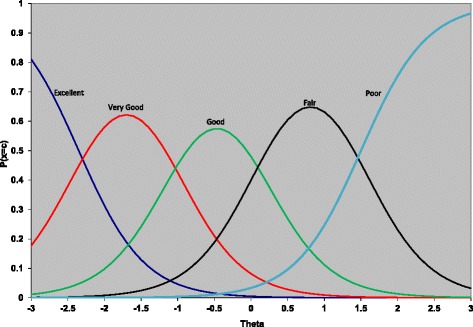
Trace line plot for the general health item from the HDCM

Figure 1 contains a graphical display of the item parameters for the general health item from the HDCM (item 1 on the CDC-PH scale) and is called a trace line or option characteristic curves. These series of curves trace the probability that an individual will choose a particular category at a particular level of the latent construct. The x-axis represents the latent construct (physical health), which is assumed to follow a standard normal distribution. As coded, higher scores indicate less health, so someone with a latent trait score of two would be said to be two standard deviations above the population (from which the sample is drawn) in terms of health problems. Each line corresponds to one of the five possible response alternatives: excellent, very good, good, fair, poor. As someone moves from left to right on the x-axis their physical health problems are increasing and we see that the category they choose moves from those indicating good physical health to those indicating physical health problems. Someone with an average level of physical health problems is about equally likely to choose “good” or “fair” as their response to the general health item. It isn’t until someone is 2.5 standard deviations below the population average of physical health problems that they predominately rate their general health as “excellent”. On the other end of the spectrum, individuals with scores of 1.5 or more are most likely to choose the “poor” category.

**Fig. 2:**
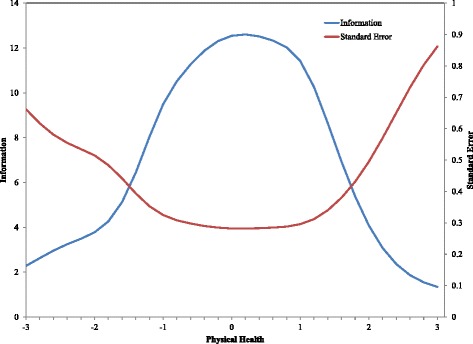
Information and standard error curves for the CDC-PH scale

In addition to the trace lines, IRT produces several summary measures related to reliability. Two of these measures, information and standard error, are most often presented graphically. Figure 2 shows the information and standard error curves for the CDC-PH scale. The standard error at any point along the x-axis is the inverse of the square root of the information at that same point on the x-axis. The metric of the information curve (located on the left hand y-axis) is difficult to interpret directly, but in a general way the scale is able to measure more precisely in regions where information is high. How precisely the scale can measure is more easily addressed using the standard error curve (matched to the right hand y-axis), which is in a standard normal metric. The x-axis in Fig. 2 is the same as in Fig. 1.

For example, a theta estimate (or IRT scale score) of 0 on the CDC-PH scale would have a standard error of 0.28. This means that a 95 % confidence interval on that participant’s score would range from −0.55 to 0.55. In contrast, consider a participant who received an IRT scale score of 2.4 (i.e., 2.4 standard deviations above the mean) on the CDC-PH scale. The information curve is much lower in this portion of the construct, which is reflected in the standard error for this score being 0.65. The same 95 % confidence interval on this participant’s score stretches over a much wider area, from 1.12 to 3.68. The less information a scale provides at a given level of theta, the less sure we can be about the accuracy of the score, which is reflected in the larger standard errors.

## Discussion

The parameter estimates for the five-item CDC-PH scale all appeared reasonable. The CDC-PH items provide reasonably reliable scores for individuals with arthritis from 1.5 standard deviations below the mean to 2 standard deviations above the mean for this latent construct. We cannot recommend using IRT with the CDC-MH scale at this time. The three items on the CDC-MH scale did not have reasonable parameter estimates. In particular, the second item, which asks about depression, has an estimated slope greater than seven thus we strongly advise against using these parameters.

To our knowledge, the CDC HRQOL measures have not been analyzed using IRT. Jiang and Hesser (2009) used the 9-items of these two Healthy Days scales (4-item HDCM and the 5-item HDSM) as indicators to assess the association between these HRQOL indicators and health risk factors [21]. Their goal was not a psychometric one and they do not discuss assessing for the IRT assumptions at all [21]. Scoring is more complex in IRT than traditional summed or proportion scores. However, given that item parameters have been obtained in this study, they do not need to be re-estimated for others to take advantage of IRT scoring procedures. IRT scoring can currently be carried out in a number of commercially available software packages, although it is expected that as more and more instruments move to computerized or web-based administration, it will become possible to use these more complex scoring algorithms without additionally burdening the end user.

Another potential limitation is the recoding scheme used with the healthy days modules. The CDC has previously proposed a recoding scheme using the following cut points: 1) 0 days, 2) 1–2 days, 3) 3–7 days, 4) > =8 days [22]. In the current data, using this recoding scheme would have resulted in over 50 % of the responses falling in the highest category causing a ceiling effect. The recoding scheme we used seems more appropriate for individuals with arthritis and these cut points alone may be an important contribution from this study.

We did not explore sensitivity to change in this cross-sectional study and future longitudinal studies should do this. As we learn more about the properties of individual items and the scales they comprise, it becomes possible to use this information when designing scales. For instance, if we knew *a priori* that a minimally important clinical difference was ½ a standard deviation then it would make sense to construct a scale capable of detecting that level of change with a desired level of accuracy. It is also possible to consider not just how big a change is of interest, but where along the construct the change occurs. Work has been done in this area assessing clinically important change in an asthma-specific HRQOL measure using Rasch modeling [23].

Our arthritis population was quite heterogeneous, including patients with established osteoarthritis or rheumatoid arthritis to those saying yes to the presence of joint symptoms in the previous month. This can be considered both a strength and a limitation of this study. Representation in the tails of a distribution can provide more data to estimate item-parameters which are related to those tails (e.g., high or low b-values). However, this can also indicate that the normality assumption for the population is not reasonable. IRT-based item parameters are related to the population from which the sample was drawn. Although there are many possible populations that would be of interest, this population has the advantage of generalizing to a broad clinical spectrum including patients from primary care settings to specialty clinics (both orthopedics and rheumatology) across a fairly diverse state. We also did not consider differential item functioning (DIF), which occurs when the relationship between items and construct(s) varies across some other variable (e.g., disease status, gender, etc.). To the extent that researchers would like to use the physical health scale to compare across different disease populations it will be important to look for DIF across these groups in future studies, as the presence of DIF can bias group comparisons [24].

## Conclusions

The analyses conducted support the feasibility of performing IRT analyses on the 5-item CDC-PH scale; and lend additional support to the notion that the CDC-PH scale is a solid measure of physical HRQOL in arthritis populations. We did not find the 3-item CDC-MH useful by itself. The results suggest that, at least in this population, an IRT approach with this scale is not advised.

## Abbreviations

HRQOL: health-related quality of life
CDC: Centers for Disease Control and Prevention
HDCM: Healthy Days Core Module
HDSM: Healthy Days Symptom Module
IRT: item response theory
NC-FM-RN: North Carolina Family Medicine Resource Network
MSK: musculoskeletal database
GRM: graded response model
CFA: confirmatory factor analysis
DWLS: diagonally weighted least squares
RMSEA: Root Mean Square Error of Approximation
CFI: Comparative Fit Index
CDC-PH: physical health scale
CDC-MH: mental health scale
